# Supplementary material for: Mega-scale experimental analysis of protein folding stability in biology and design
Source: Nature. 2023 Jul 19;620(7973):434–44. doi: 10.1038/s41586-023-06328-6 (PMC10412457; doi:10.1038/s41586-023-06328-6)
Supplement: Supplementary file 1 — This file includes three supplementary tables, eight supplementary figures and notes. [file 41586_2023_6328_MOESM1_ESM.pdf]

nature portfolio

https://doi.org/10.1038/s41586-023-06328-6

Supplementary information

Mega-scale experimental analysis of protein folding stability in biology and design

In the format provided by the authors and unedited

1. Supplementary Tables

Supplementary Table 1 Condition for measuring folding stability in the previous papers.

Protein	PDB ID	Pubmed ID	Reference	Offset in Fig.1G (kcal/mol)	Temperature (°C)	Buffer	pH	Numbering (Ours-Ref)
Protein GB1	1PGA	31371509	[29]	-0.8	25	NaPi	6.5	0
NTL9	2HBB	28494951	[59]	1.9	25	20 mM NAOAc, 100 mM NaCl	5.5	0
SAP domain	2WQG	26073259	[60]	1.2	10	50 mM MES pH 6.0, 500 mM NaCl	6	-1
hPin1 WW domain	2M8I	19565466	[61]	-1.9	40	10 mM NaPi	7	0
hYap65 WW domain	1K9Q	11420447	[62]	-2.3	60	20 mM KPi, 100 mM NaCl	7	0
hYap65 WW domain	1K9Q	23035249	[63]	-2.3	50	20 mM NaPi	7	0
Villin HP35	1VII	23798426/19354264	[64,65]	0.1	25	10 mM NaOAc, 150 mM NaCl	5	0
BBL	2WAV	19445954	[66]	0.3	10	50 mM KPi 200 mM KCl	7	0
FF domain	1UZC	15935381	[67]	0.8	10	50 mM NAOAc, 100 mM NaCl	5.7	-7
ADA2h activation domain	1O6X	9799641	[68]	-0.2	25	50 mM NaPi	7	-7
hFyn SH3 domain	1SHF	9819209/12079394/16142914	[69–71]	-0.1	25	10 mM Tris, 0.2 mM EDTA and 250 mM KCl	8	0

Supplementary Table 2 Description of metrics in Supplementary Fig. 2

Metrics	Description
NA_frac	Fraction of data without sufficient NGS reads
dg_corr	Correlation between ΔG from trypsin challenge and chymotrypsin challenge
slope	Slope of linear fitting line between ΔG from trypsin challenge and chymotrypsin challenge
y_intercept	Y-intercept of linear fitting line between ΔG from trypsin challenge and chymotrypsin challenge
wt_dg_med	Median ΔG of WT
frac_pos_with_hydrophobic_stabilzing_muts	Fraction of stabilizing mutations which replace hydrophobic amino acids
wt_k50_std_max	Standard deviation of WT ΔG
wt_k50_diff_max	Max-min of WT ΔG
wt_d_from_line	Distance between WT data points and linear fitting line from mutational data
num_cys	The number of cysteins
cut_potential_k50_max	Cleavage potential in folded state calculated by K_{50}
cut_potential_dG_max	Cleavage potential in folded state calculated by ΔG

Supplementary Table 3 Description of columns in Tsuboyama2023_Dataset2_Dataset3_20230416.csv

Column name	Description
name	sequence name
dna_seq	DNA sequence
log10_K50_t	Median of posteriors of K_{50} trypsin in log10 scale (μM)
log10_K50_t_95CI_high	Top 2.5%ile of posterior of K_{50} trypsin in log10 scale (μM)
log10_K50_t_95CI_low	Top 97.5%ile of posterior of K_{50} trypsin in log10 scale (μM)
log10_K50_t_95CI	log10_K50_t_95CI_high - log10_K50_t_95CI_low
fitting_error_t	Absolute error between the observed counts and the expected counts for a given sequence (based on all model parameters related to trypsin data), averaged over 24 conditions and normalized by the observed counts in the no-protease samples for that sequence
log10_K50unfolded_t	K_{50} unfolded trypsin in log10 scale (μM)
deltaG_t	ΔG calculated from log10_K50_t (kcal/mol)
deltaG_t_95CI_high	ΔG calculated from log10_K50_t_95CI_high (kcal/mol)
deltaG_t_95CI_low	ΔG calculated from log10_K50_t_95CI_low (kcal/mol)
deltaG_t_95CI	deltaG_t_95CI_high - deltaG_t_95CI_low (kcal/mol)
log10_K50_c	Median of posteriors of K_{50} chymotrypsin in log10 scale (μM)
log10_K50_c_95CI_high	Top 2.5%ile of posterior of K_{50} chymotrypsin in log10 scale (μM)
log10_K50_c_95CI_low	Top 97.5%ile of posterior of K_{50} chymotrypsin in log10 scale (μM)
log10_K50_c_95CI	log10_K50_c_95CI_high - log10_K50_c_95CI_low
fitting_error_c	Absolute error between the observed counts and the expected counts for a given sequence (based on all model parameters related to chymotrypsin data), averaged over 24 conditions and normalized by the observed counts in the no-protease samples for that sequence
log10_K50unfolded_c	K_{50} unfolded chymotrypsin in log10 scale (μM)
deltaG_c	ΔG calculated from log10_K50_c (kcal/mol)
deltaG_c_95CI_high	ΔG calculated from log10_K50_c_95CI_high (kcal/mol)

deltaG_c_95CI_low	ΔG calculated from log10_K50_c_95CI_low (kcal/mol)
deltaG_c_95CI	deltaG_c_95CI_high - deltaG_c_95CI_low (kcal/mol)
deltaG	Median of posterior of ΔG from trypsin+chymotrypsin data (kcal/mol)
deltaG_95CI_high	Top 2.5%ile posterior of ΔG from trypsin+chymotrypsin data (kcal/mol)
deltaG_95CI_low	Top 97.5%ile posterior of ΔG from trypsin+chymotrypsin data (kcal/mol)
deltaG_95CI	deltaG_95CI_high - deltaG_95CI_low
aa_seq_full	Amino acid sequence including padding linker sequence
aa_seq	Amino acid sequence without linker sequence
mut_type	Mutation type (like WT, substitution, insertion, deletion, or double mutants)
WT_name	Name of wild-type domain
WT_cluster	Cluster number of wild-type domain
log10_K50_trypsin_ML	K_{50} trypsin in log10 scale for machine learning (μM)<br>('-' means lacking or unreliable data)
log10_K50_chymotrypsin_ML	K_{50} chymotrypsin in log10 scale for machine learning (μM)<br>('-' means lacking or unreliable data)
dG_ML	ΔG for machine learning (kcal/mol) ('-' means unreliable data)
ddG_ML	ΔΔG for machine learning (kcal/mol)<br>('-' means unreliable data)
Stabilizing_mut	True if ΔΔG>1 (kcal/mol) and ΔΔG is reliable
name_original	Names we originally used
pair_name	Amino acid pair name for double mutants, NA for WT or single mutants

2. Supplementary Discussion

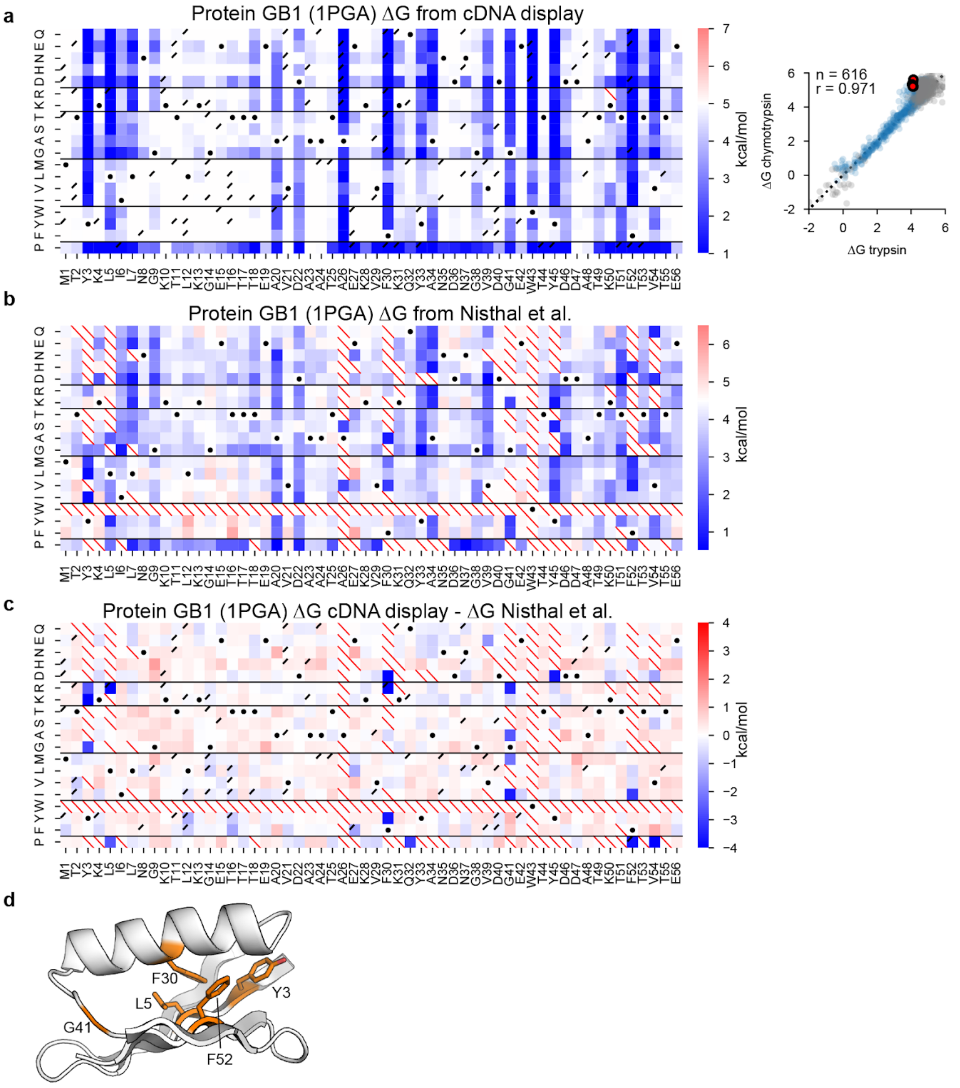

Supplementary Fig. 1 Folding stability discrepancies between cDNA display proteolysis and previous measurements on Protein GB1

(a) Left: Mutational scanning results from cDNA display proteolysis. As in Fig. 2, white represents the folding stability of wild-type and red/blue indicates stabilizing/destabilizing mutations. Black dots indicate the wild-type amino acid, red slashes indicate missing data, and black corner slashes indicate lower confidence ΔG estimates, (95% confidence interval > 0.5 kcal/mol), including ΔG estimates near the edges of the dynamic range. Right: Agreement between variant ΔG values independently determined using assays with trypsin (x-axis) and chymotrypsin (y-axis). Multiple codon variants of the wild-type sequence are shown in red,

reliable ΔG values in blue, and less reliable ΔG estimates (same as above) in gray. The black dashed lines represent Y=X. Each plot shows the number of reliable points and the Pearson r-value.

(b) Mutational scanning results from robotics-enabled high-throughput purification and chemical denaturation[29], colored as in (a).

(c and d) Difference heat-map (c) showing the consistency between cDNA display proteolysis (a) and robotics-enabled high-throughput purification and chemical denaturation (b). Dark blue squares indicate highly inconsistent positions where cDNA display proteolysis (a) observes low stability but robotics-assisted chemical denaturation (b) observes high stability. In many cases, the chemical denaturation data indicates that polar substitutions into the hydrophobic core are relatively tolerated (e.g. Y3K, L5R, F30D, F30R, Y45D), whereas cDNA display proteolysis indicates that these substitutions are very destabilizing.

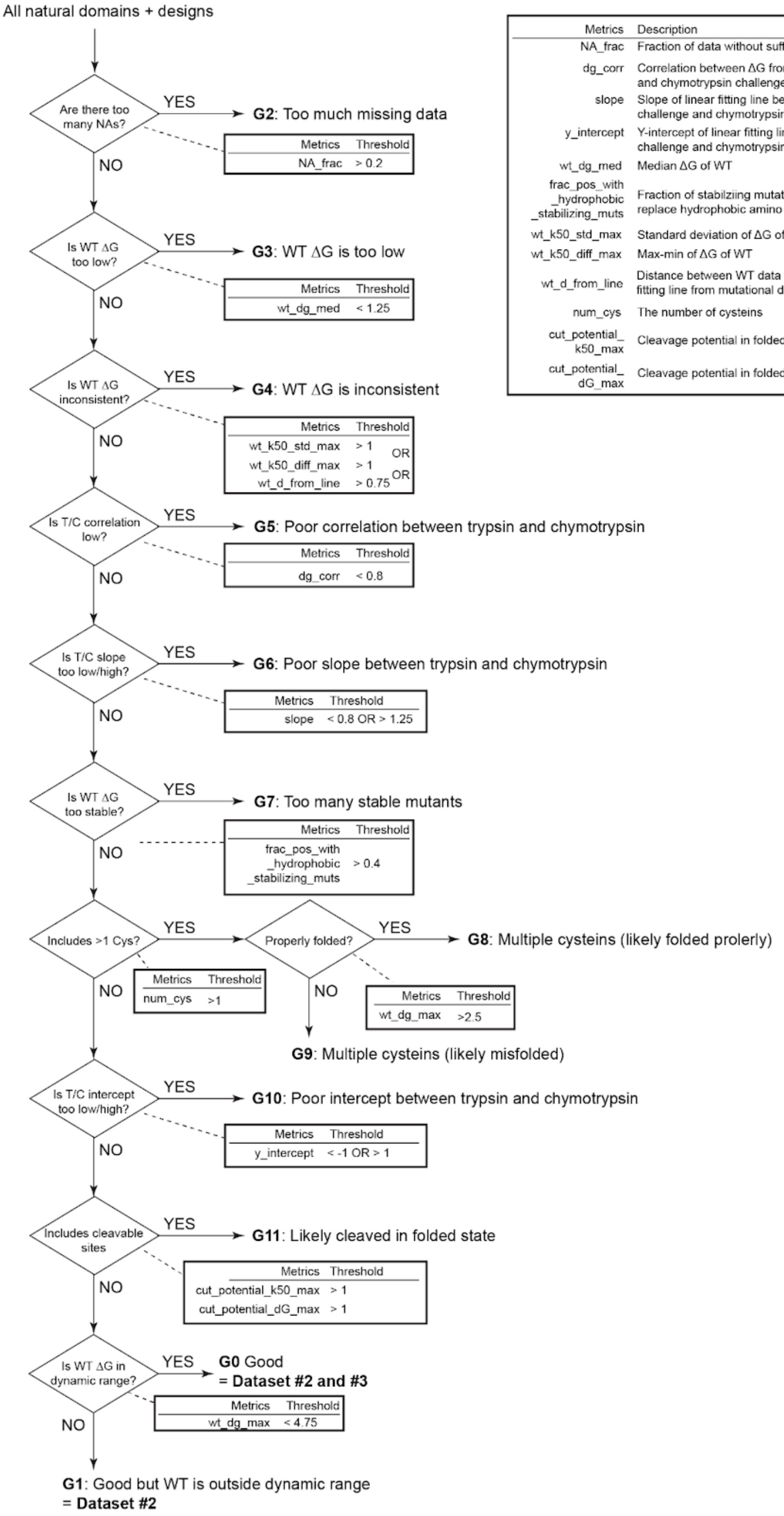

Metrics	Description
NA_frac	Fraction of data without sufficient NGS reads
dg_corr	Correlation between ΔG from trypsin challenge and chymotrypsin challenge
slope	Slope of linear fitting line between ΔG from trypsin challenge and chymotrypsin challenge
y_intercept	Y-intercept of linear fitting line between ΔG from trypsin challenge and chymotrypsin challenge
wt_dg_med	Median ΔG of WT
frac_pos_with _hydrophobic _stabilizing_muts	Fraction of stabilziing mutations which replace hydrophobic amino acids
wt_k50_std_max	Standard deviation of ΔG of WT
wt_k50_diff_max	Max-min of ΔG of WT
wt_d_from_line	Distance between WT data points and linear fitting line from mutational data
num_cys	The number of cysteins
cut_potential_ k50_max	Cleavage potential in folded state calculated by K_{50}
cut_potential_ dG_max	Cleavage potential in folded state calculated by ΔG

Supplementary Fig. 2 Classification of deep mutational scanning results

We classified all deep mutational scanning results into nine groups shown in Fig. 2b. Here, we show the classification criteria. The description of all metrics is also included in Supplementary Table 2, and the metrics of all domains for the classification are included in Single_DMS_list.csv.

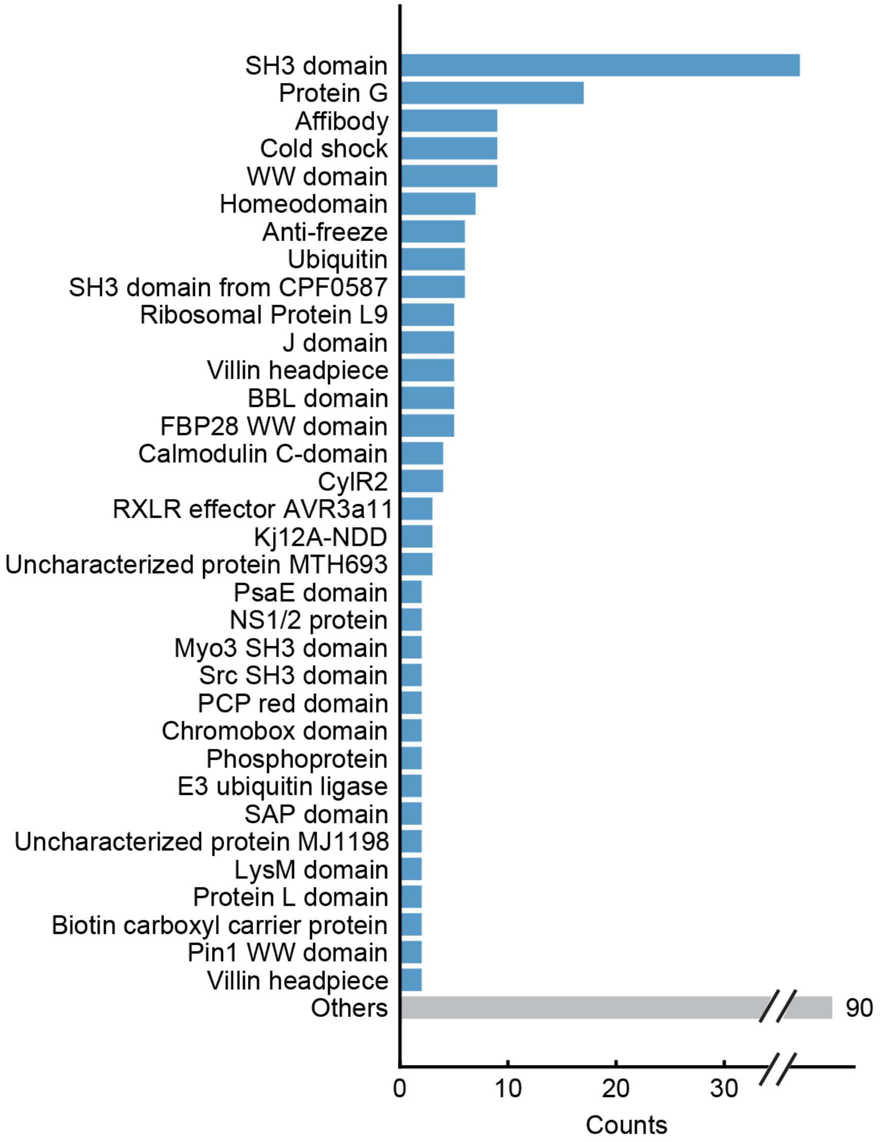

Supplementary Fig. 3 Classification of the natural protein domains investigated in cDNA display proteolysis

Comprehensive group list of wild-type structures classified as G0 in Fig. 2b grouped into domain families.

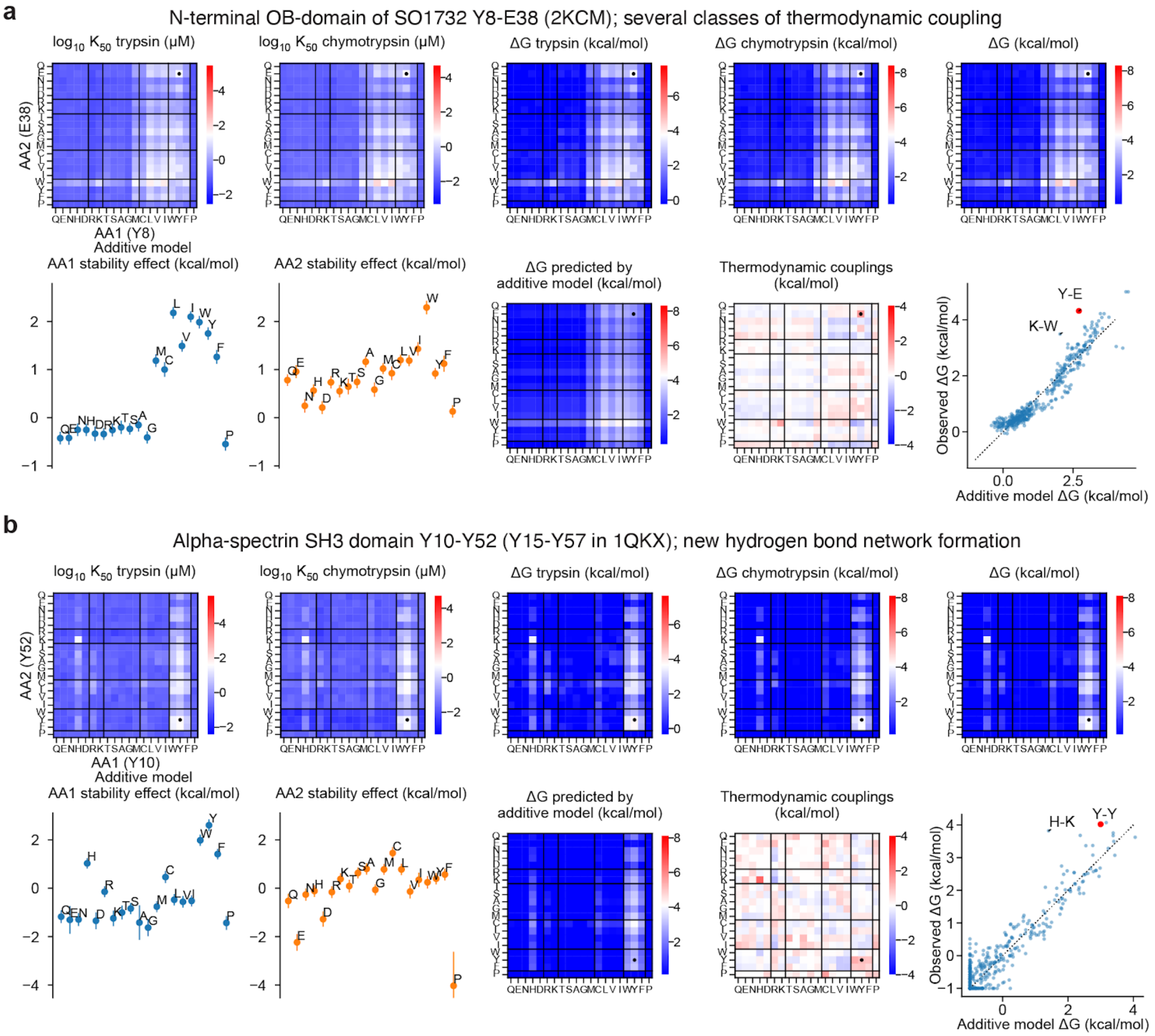
a
N-terminal OB-domain of SO1732 Y8-E38 (2KCM); several classes of thermodynamic coupling
log10 K50 trypsin (μM)
log10 K50 chymotrypsin (μM)
ΔG trypsin (kcal/mol)
ΔG chymotrypsin (kcal/mol)
ΔG (kcal/mol)
AA2 (E38)
AA1 (Y8)
Additive model
AA1 stability effect (kcal/mol)
AA2 stability effect (kcal/mol)
ΔG predicted by additive model (kcal/mol)
Thermodynamic couplings (kcal/mol)
Y-E
K-W
Observed ΔG (kcal/mol)
Additive model ΔG (kcal/mol)
b
Alpha-spectrin SH3 domain Y10-Y52 (Y15-Y57 in 1QKX); new hydrogen bond network formation
log10 K50 trypsin (μM)
log10 K50 chymotrypsin (μM)
ΔG trypsin (kcal/mol)
ΔG chymotrypsin (kcal/mol)
ΔG (kcal/mol)
AA2 (Y52)
AA1 (Y10)
Additive model
AA1 stability effect (kcal/mol)
AA2 stability effect (kcal/mol)
ΔG predicted by additive model (kcal/mol)
Thermodynamic couplings (kcal/mol)
H-K
Y-Y
Observed ΔG (kcal/mol)
Additive model ΔG (kcal/mol)

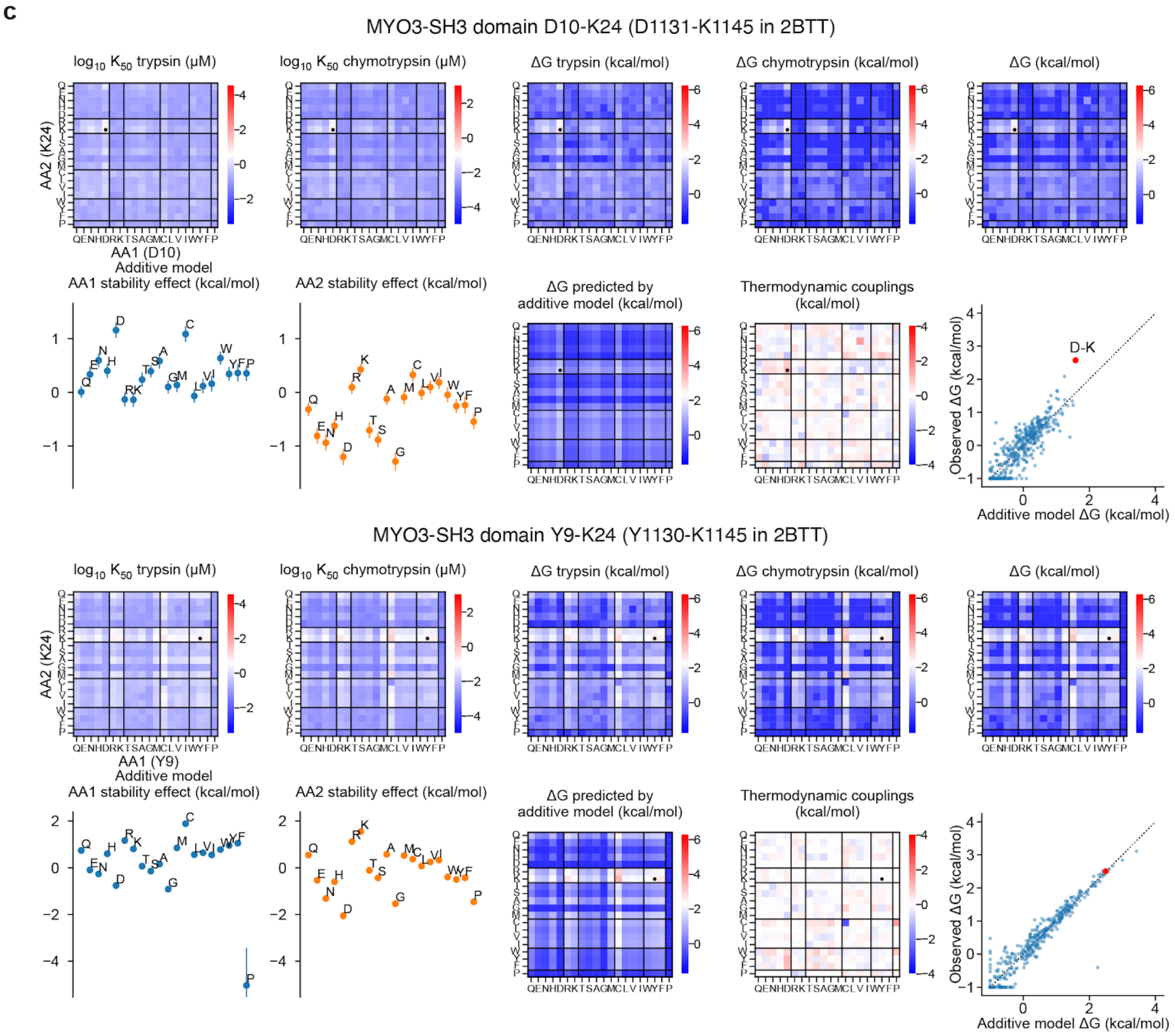
c
MYO3-SH3 domain D10-K24 (D1131-K1145 in 2BTT)
log10 K50 trypsin (μM)
log10 K50 chymotrypsin (μM)
ΔG trypsin (kcal/mol)
ΔG chymotrypsin (kcal/mol)
ΔG (kcal/mol)
AA2 (K24)
AA1 (D10)
Additive model
AA1 stability effect (kcal/mol)
AA2 stability effect (kcal/mol)
ΔG predicted by additive model (kcal/mol)
Thermodynamic couplings (kcal/mol)
D-K
Observed ΔG (kcal/mol)
Additive model ΔG (kcal/mol)
MYO3-SH3 domain Y9-K24 (Y1130-K1145 in 2BTT)
log10 K50 trypsin (μM)
log10 K50 chymotrypsin (μM)
ΔG trypsin (kcal/mol)
ΔG chymotrypsin (kcal/mol)
ΔG (kcal/mol)
AA2 (K24)
AA1 (Y9)
Additive model
AA1 stability effect (kcal/mol)
AA2 stability effect (kcal/mol)
ΔG predicted by additive model (kcal/mol)
Thermodynamic couplings (kcal/mol)
Observed ΔG (kcal/mol)
Additive model ΔG (kcal/mol)

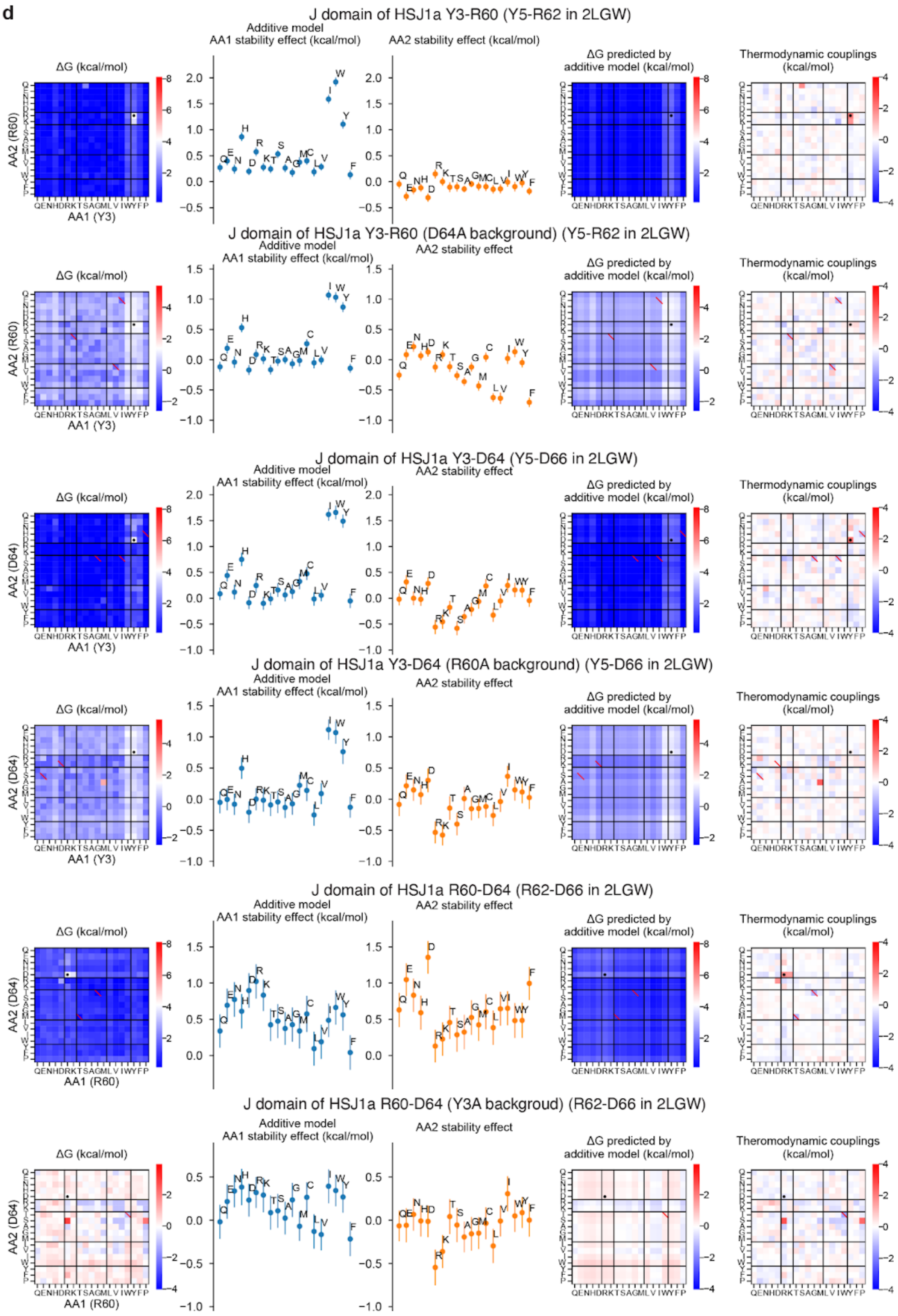
d
J domain of HSJ1a Y3-R60 (Y5-R62 in 2LGW)
Additive model
AA1 stability effect (kcal/mol)
AA2 stability effect (kcal/mol)
ΔG (kcal/mol)
ΔG predicted by additive model (kcal/mol)
Thermodynamic couplings (kcal/mol)
AA2 (R60)
AA1 (Y3)
J domain of HSJ1a Y3-R60 (D64A background) (Y5-R62 in 2LGW)
AA2 stability effect
J domain of HSJ1a Y3-D64 (Y5-D66 in 2LGW)
AA2 (D64)
J domain of HSJ1a Y3-D64 (R60A background) (Y5-D66 in 2LGW)
Theromodynamic couplings (kcal/mol)
J domain of HSJ1a R60-D64 (R62-D66 in 2LGW)
AA1 (R60)
J domain of HSJ1a R60-D64 (Y3A backgroud) (R62-D66 in 2LGW)
QENHDRKTSAGMLVIWYFP

Supplementary Fig. 4 Comprehensive double mutational data for the notable amino acid pairs

(a and b) Analysis of thermodynamic coupling for two notable amino acid pairs. In the first row, we show stabilities for all 20x20 double mutants according to five different experimental metrics. From left to right, we show trypsin K_{50}, chymotrypsin K_{50}, ΔG inferred from trypsin experiments, ΔG inferred from chymotrypsin experiments, and ΔG inferred from both sets of experiments together. In the second row, we show the results of the additive model. From left to right, the first two plots show the inferred single amino acid terms for all 20 amino acids in the first and second sites of the amino acid pair. Error bars represent the standard deviation of the posterior distributions (n=25) . The middle heatmap shows stability (ΔG) for all amino acid pairs according to the additive model (the sum of the two single amino acid terms). The fourth plot shows the observed thermodynamic coupling; e.g. the experimental ΔG (rightmost plot in the first row) minus the prediction from the additive model (middle plot of the second row). The final scatter plot shows experimental stabilities for all double mutants (y-axis) plotted against the results from the additive model (x-axis).

(c) Same analysis as (a) and (b) for two site pairs in MYO3-SH3 domain (2BTT).

(d) Analysis of thermodynamic coupling for all amino acid pairs from a notable amino acid triple. The same amino acid substitutions were also performed for the mutant background with the third amino acid replaced by Ala. From left to right, we show the stabilities (ΔG) of all pairs of amino acids, the single amino acid terms in the additive model (error bars show the standard deviation of the posterior distribution), the stabilities for all pairs according to the additive model, and the thermodynamic coupling for all pairs of amino acids.

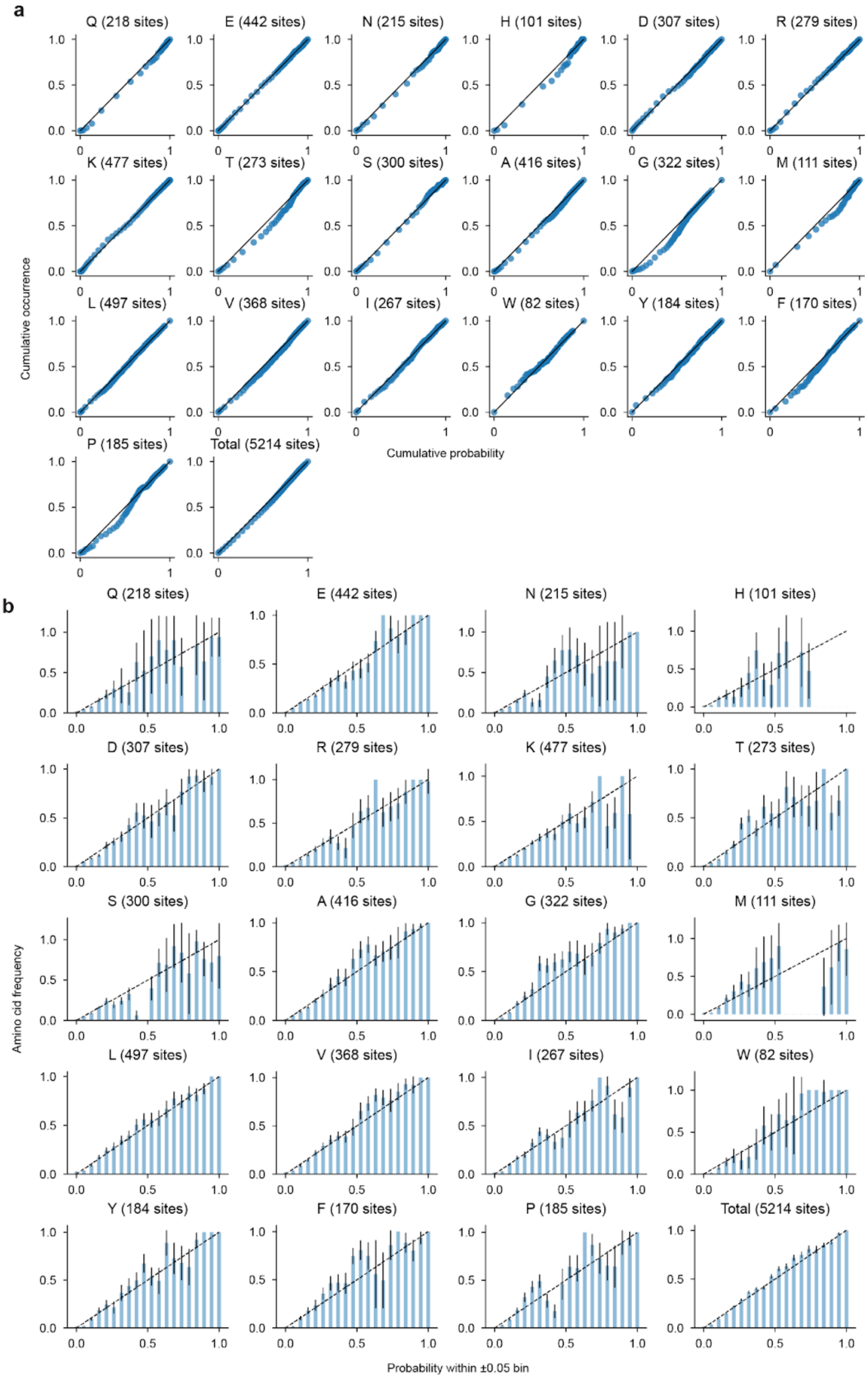
a
Q (218 sites)
E (442 sites)
N (215 sites)
H (101 sites)
D (307 sites)
R (279 sites)
K (477 sites)
T (273 sites)
S (300 sites)
A (416 sites)
G (322 sites)
M (111 sites)
L (497 sites)
V (368 sites)
I (267 sites)
W (82 sites)
Y (184 sites)
F (170 sites)
P (185 sites)
Total (5214 sites)
Cumulative occurrence
Cumulative probability
b
Q (218 sites)
E (442 sites)
N (215 sites)
H (101 sites)
D (307 sites)
R (279 sites)
K (477 sites)
T (273 sites)
S (300 sites)
A (416 sites)
G (322 sites)
M (111 sites)
L (497 sites)
V (368 sites)
I (267 sites)
W (82 sites)
Y (184 sites)
F (170 sites)
P (185 sites)
Total (5214 sites)
Amino cid frequency
Probability within ±0.05 bin

Supplementary Fig. 5 Testing calibration of classification model for predicting wild-type amino acids

(a) Relationship between predicted cumulative probability and observed cumulative occurrence for each of 19 amino acids and total data. For each of the 19 amino acids (excluding Cys), we order all 4,718 sites from lowest to highest probability for that amino acid, then step through the sites in that order while plotting the fraction of the total cumulative probability (x-axis) and the fraction of all occurrences of that amino acid (y-axis). For the "Total" plot, we order all 89,642 (4,718*19) amino acid possibilities at all sites from lowest probability to highest probability, then step through all amino acid possibilities in that order while plotting the fraction of the total cumulative probability (x-axis) and the fraction of all actual amino acid occurrences (y-axis). The black diagonal lines show Y=X.

(b) Relationship between modeled amino acid probabilities and actual amino acid frequencies. For each of the 19 amino acids (excluding Cys), we binned all 4,718 sites into 20 bins according to the probability of that amino acid. Bins are spaced every 0.05 probability units and each bin has a width of 0.1, so sites can appear in two neighboring bins. For each bin (x-axis), the bar shows the true frequency of that amino acid in that bin (y-axis); error bars indicate the standard deviation of the true frequency from bootstrap resampling of all the sites. The black diagonal lines show Y=X (e.g. the predicted probability matches the true frequency). For the "Total" plot, we binned all 89,642 (4,718*19) amino acid possibilities at all sites as before, then counted the fraction of matching amino acids in each bin. Error bars represent the standard deviation of the frequencies from bootstrap resampling of all sites (n=50) .

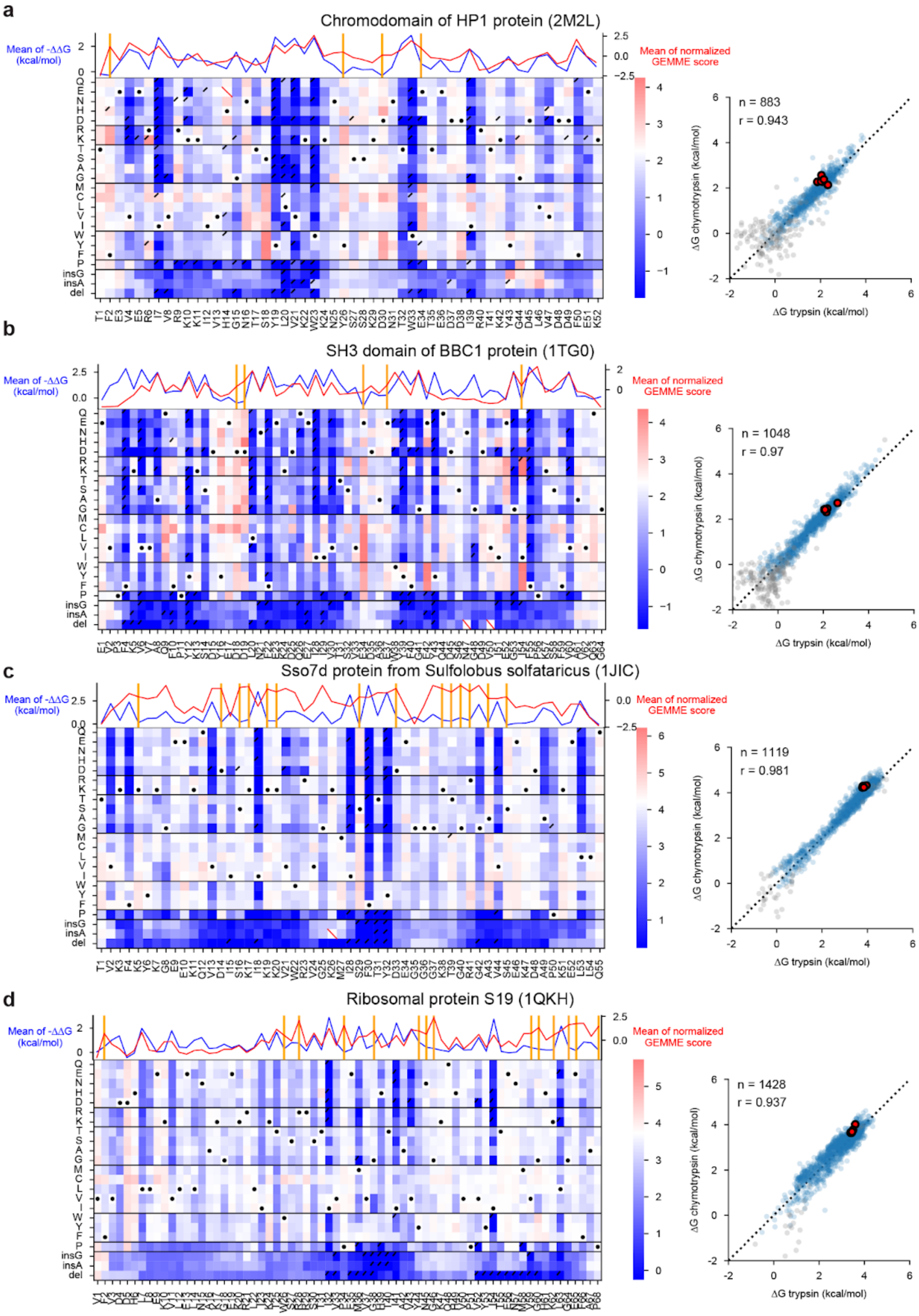

a
Chromodomain of HP1 protein (2M2L)
Mean of -ΔΔG (kcal/mol)
Mean of normalized GEMME score
n = 883
r = 0.943
ΔG chymotrypsin (kcal/mol)
ΔG trypsin (kcal/mol)
b
SH3 domain of BBC1 protein (1TG0)
Mean of -ΔΔG (kcal/mol)
Mean of normalized GEMME score
n = 1048
r = 0.97
ΔG chymotrypsin (kcal/mol)
ΔG trypsin (kcal/mol)
c
Sso7d protein from Sulfolobus solfataricus (1JIC)
Mean of -ΔΔG (kcal/mol)
Mean of normalized GEMME score
n = 1119
r = 0.981
ΔG chymotrypsin (kcal/mol)
ΔG trypsin (kcal/mol)
d
Ribosomal protein S19 (1QKH)
Mean of -ΔΔG (kcal/mol)
Mean of normalized GEMME score
n = 1428
r = 0.937
ΔG chymotrypsin (kcal/mol)
ΔG trypsin (kcal/mol)

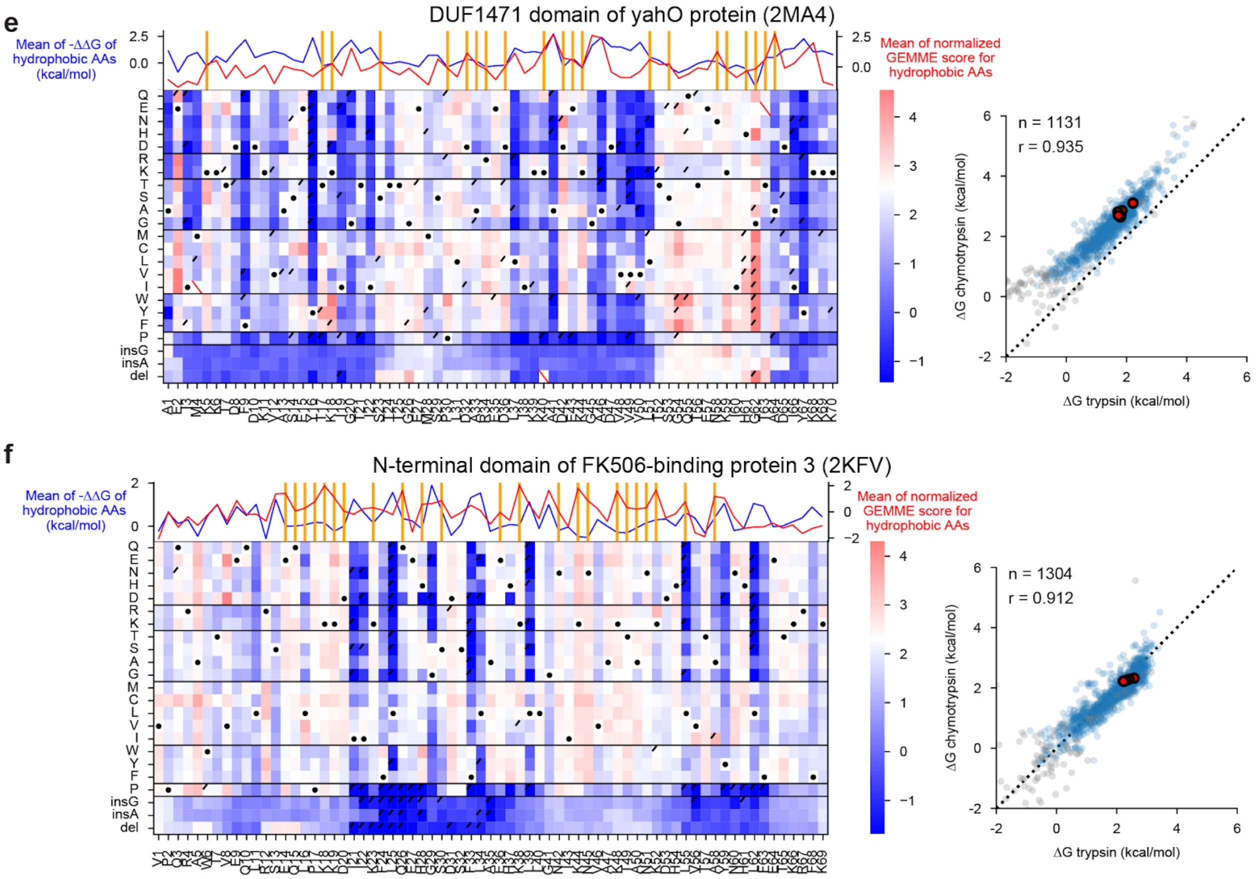

Supplementary Fig. 6 Heat maps for notable domains with functional residues

(a-d) Mutational scanning results for four domains. Left: Heat maps show ΔG for substitutions, deletions, and Gly and Ala insertions at each residue. White indicates the wild-type stability and red/blue indicates stabilizing/destabilizing. Black dots indicate the wild-type amino acid, red slashes indicate missing data, and black corner slashes indicate lower confidence ΔG estimates, (95% confidence interval > 0.5 kcal/mol), including ΔG estimates near the edges of the dynamic range. At top, lines show the mean ΔΔG (blue) and the mean normalized GEMME score (red), with functional sites (classified according to Extended Data Fig. 9a) marked with vertical orange lines. Right: Agreement between variant ΔG values independently determined using assays with trypsin (x-axis) and chymotrypsin (y-axis). Multiple codon variants of the wild-type sequence are shown in red, reliable ΔG values in blue, and less reliable ΔG estimates (same as above) in gray. The black dashed line represents Y=X. Each plot shows the number of reliable points and the Pearson r-value for the blue (reliable) points.

(e-f) Same as (a-d), but top lines indicate the mean of ΔΔG for hydrophobic amino acid substitutions (blue) and mean normalized GEMME score of hydrophobic amino acids (red). Functional sites are classified according to Extended Data Fig. 9g.

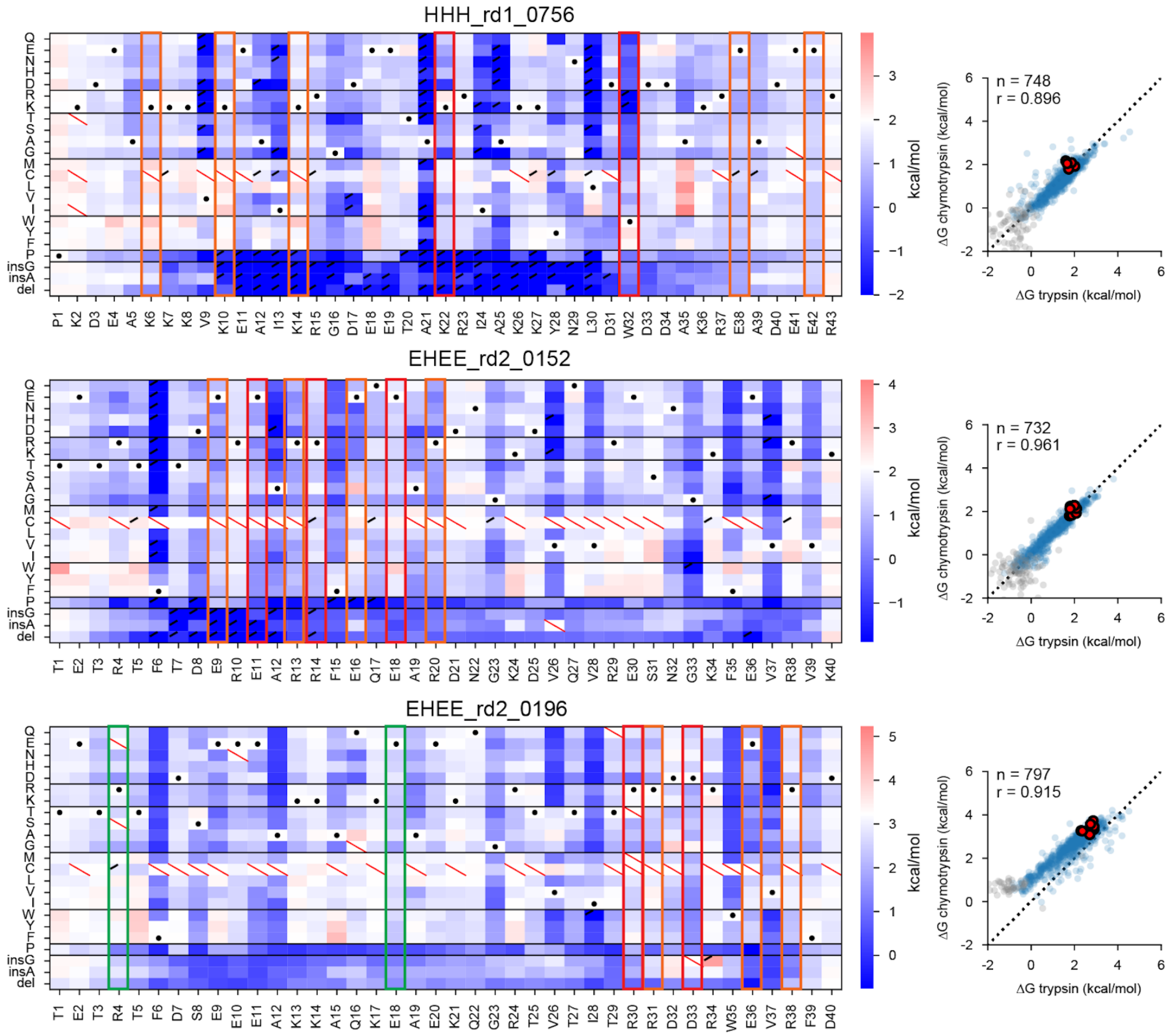

Supplementary Fig. 7 Heat maps for three designed domains with notable polar interactions.

Mutational scanning results for three domains with notable polar interactions. Left: Heat maps show ΔG for substitutions, deletions, and Gly and Ala insertions at each residue. White indicates the wild-type stability and red/blue indicates stabilizing/destabilizing. Black dots indicate the wild-type amino acid, red slashes indicate missing data, and black corner slashes indicate lower confidence ΔG estimates, (95% confidence interval > 0.5 kcal/mol), including ΔG estimates near the edges of the dynamic range. The polar networks shown in Extended Data Fig. 10b are highlighted in orange, red, and green. Right: Agreement between variant ΔG values independently determined using assays with trypsin (x-axis) and chymotrypsin (y-axis). Multiple codon variants of the wild-type sequence are shown in red, reliable ΔG values in blue, and less reliable ΔG estimates (same as above) in gray. The black dashed line represents Y=X. Each plot shows the number of reliable points and the Pearson r-value for the blue (reliable) points

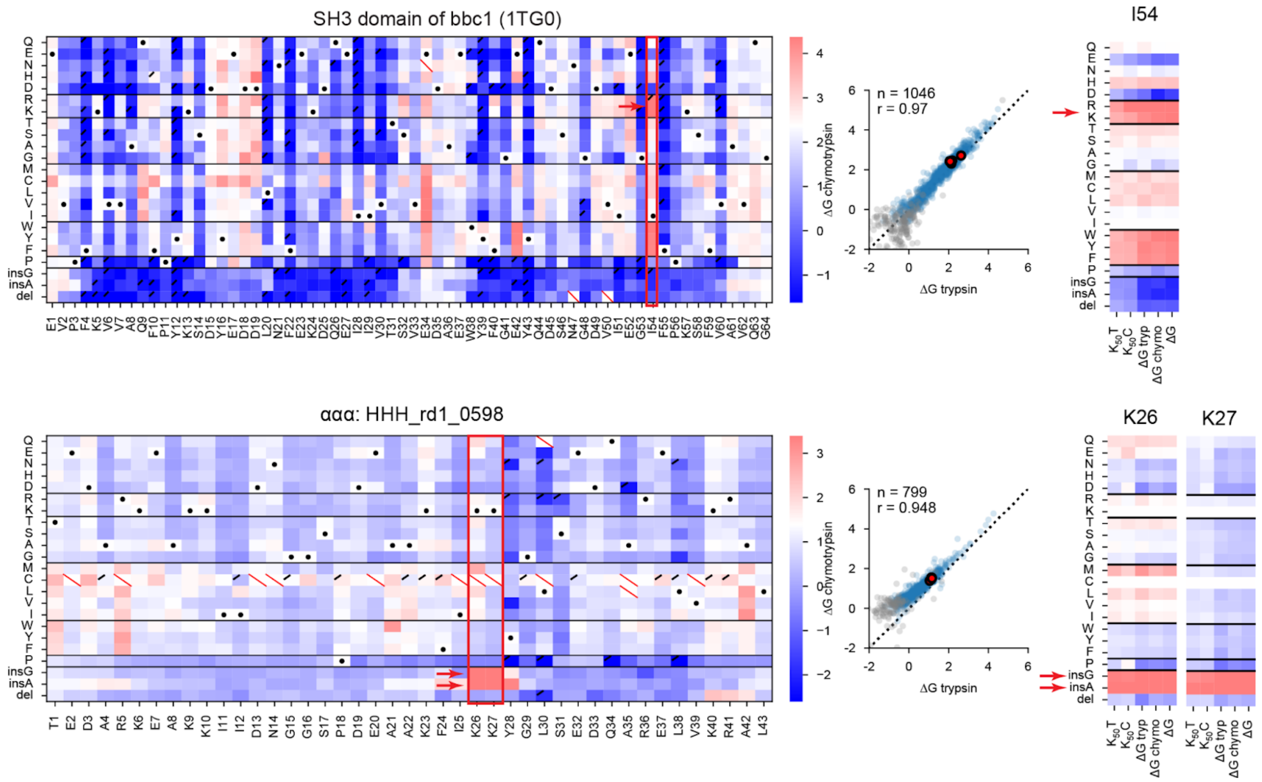

Supplementary Fig. 8 Heat maps for three domains with notable stabilizing mutations

Left: Heat maps show ΔG for substitutions, deletions, and Gly and Ala insertions at each residue. White indicates the wild-type stability and red/blue indicates stabilizing/destabilizing. Black dots indicate the wild-type amino acid, red slashes indicate missing data, and black corner slashes indicate lower confidence ΔG estimates, (95% confidence interval > 0.5 kcal/mol), including ΔG estimates near the edges of the dynamic range. The red boxes and arrows highlight sites with notable stabilizing mutations. Middle: Agreement between variant ΔG values independently determined using assays with trypsin (x-axis) and chymotrypsin (y-axis). Multiple codon variants of the wild-type sequence are shown in red, reliable ΔG values in blue, and less reliable ΔG estimates (same as above) in gray. The black dashed line represents Y=X. Each plot shows the number of reliable points and the Pearson r-value for the blue (reliable) points. Right: For four positions with stabilizing mutations, heatmaps show five experimental metrics: the trypsin (T) and chymotrypsin (C) K_{50} values, the ΔG values inferred from trypsin and chymotrypsin experiments, and the overall ΔG inferred from both trypsin and chymotrypsin experiments together.

3. Supplementary Notes

Structure of the K_{50} model to infer K_{50} values from next-generation sequencing data

We modeled our selection results using the single turnover kinetics model described in Fig. 1b. We chose this model because we expect that the total concentration of protein-cDNA complex is low compared to the amount of added enzyme and because the model captures the saturation behavior observed by qPCR at high enzyme concentration (Extended Data Fig. 1). Instead of attempting to capture the microscopic complexity of our system (millions of different substrates and potential inhibitors), the purpose of the model is to treat each substrate in a consistent, simplified manner and infer reasonable parameters.

Our model makes two main assumptions. First, we assume that each sequence is cleaved independently, with no competition or product inhibition. As described by Fig. 1 eqs. 2 and 3, cleavage is described by four parameters: enzyme concentration (*E)*, time (*t*), and the kinetic parameters K_{50} and $k_{\max}$. All experiments used a fixed five minute reaction time. Based on qPCR analysis of individual sequences (Extended Data Fig. 1), we fixed the quantity $k_{max} * t$ at $10^{0.65}$ for all sequences. Each sequence's unique stability is defined by the K_{50} parameter that represents the enzyme concentration producing the half maximal cleavage rate (Fig. 1b eq. 3). Our second main assumption is that we can interpret our K_{50} values as representing the dissociation constants (K_D) between each protein sequence and the enzyme ($K_{50} \approx K_D$, Fig. 1b eq. 6). From this assumption, we can determine the folding stability of each sequence (ΔG) based on the relationship between the observed K_{50} value and theoretical K_{50} values for the fully folded and fully unfolded states ($K_{50,F}$ and $K_{50,U}$, Fig. 1b eqs. 5-7). Although we can directly fit K_{50} values without making any assumptions about the microscopic basis for K_{50} (see Supplementary information for the detail), assuming that $K_{50} \approx K_D$ aids our interpretation and enables us to directly fit ΔG values to our data using the *Coupled* approach described below.

To fit our model to our sequencing counts data, we first assume that the cDNA display process produces an unknown initial distribution of full-length protein-cDNA complexes (the $cDNA_0$ distribution). The distribution of sequences at enzyme concentration E (the $cDNA_E$ distribution) is the product of the initial sequence distribution $cDNA_0$ and the surviving fraction of each sequence according to Fig. 1 eqs. 2 and 3, after re-normalizing the total surviving fraction of all sequences to 1.

$$cDNA_{E,i} = cDNA_{0,i} * Frac\ ([E], K_{50,i}) / \sum_j (cDNA_{0,j} * Frac\ ([E], K_{50,j}) \quad (8)$$

Finally, we assume that our deep sequencing counts result from n_{sel} independent selections from the $cDNA_E$ distribution, where n_{sel} is the number of sequencing reads that exactly matched our specified DNA sequences.

We apply the K_{50} model in two different ways based on whether K_{50} values for trypsin and chymotrypsin are *Independent* or *Coupled*. The "Independent" procedure is used in Steps 1, 2

and 5 in the section “Procedure for fitting all data”. In the independent procedure, the inputs to the model are the sequencing counts data from experiments with one protease, the enzyme concentrations, the reaction time, and the k_{max} constant. We fit the model by sampling two parameters per sequence from normal prior distributions: (1) K_{50}, and (2) the initial fraction of each sequence in the $cDNA_0$ distribution. The “Coupled” procedure is used in Step 5 in the section “Procedure for fitting all data”. In the coupled procedure, the inputs to the model are the sequencing counts data from experiments with both proteases, the enzyme concentrations, the reaction time, the k_{max} constant, the $K_{50,F}$ constants representing the universal K_{50} value for sequences in the folded state (one for each protease), and the predicted $K_{50,U}$ values for all sequences for both proteases from the unfolded state model. We then assume that each sequence has a specific ΔG value that is shared across both proteases. We use this shared ΔG value along with $K_{50,F}$ and $K_{50,U}$ (for each protease) to determine K_{50} for each protease according to Fig. 1 Eqs. 5 and 7. Finally, we fit the coupled model by sampling two parameters per sequence from normal prior distributions: (1) ΔG, and (2) the initial fractions of each sequence in $cDNA_0$.

Full results from both the independent and coupled fitting procedure are provided in Tsuboyama2023_Dataset2_Dataset3_20230416.csv. For our stability parameters (protease-specific K_{50} in the independent procedure and ΔG in the coupled procedure) we report the median of the posterior distribution as well as the upper and lower limits of the 95% confidence interval (the 2.5%ile and 97.5%ile values of the posterior distribution). We also used the protease-specific K_{50} values from the independent procedure to compute protease-specific ΔG values. We do this using the same $K_{50,F}$ and $K_{50,U}$ values used in the coupled procedure according to Fig. 1 Eqs. 5 and 7. These protease-specific ΔG estimates are also reported in Tsuboyama2023_Dataset1_20230416.csv and are only used to examine the consistency between different proteases (e.g. Fig. 1f and Fig. 2d). In some cases, the independently fit K_{50} values can lead to impossible values for ΔG. This can occur if K_{50} is higher than $K_{50,F}$ (observed cleavage is slower than our limit for cleavage in the folded state) or if K_{50} is lower than $K_{50,U}$ (observed cleavage is faster than predicted cleavage in the unfolded state). If the median protease-specific K_{50} or the confidence interval limits for a particular sequence lead to impossible ΔG values for that sequence, we report dummy values for the corresponding protease-specific ΔG estimates.

Structure of the unfolded state model to infer unfolded K_{50} ($K_{50,U}$) from scrambled sequence data

Our unfolded state model is similar to the model employed previously[18] with two notable differences. First, instead of assuming that all scrambled sequences are fully unfolded, we assume that each scrambled sequence has its own unknown folding stability, with a prior distribution biased toward low stability (normal prior centered at ΔG = -1, sigma = 4). Second, instead of fitting an unfolded state model for each protease independently, we assume that each scrambled sequence’s stability (ΔG) is common across both proteases, and fit the models for each protease together. As a result, the majority of scrambled sequences are modeled as completely unfolded (Extended Data Fig. 2c), but some scrambled sequences are modeled as stable when that interpretation is consistent with both the trypsin and chymotrypsin data.

Our unfolded state has three parts: (1) a position specific scoring matrix (PSSM) that describes how the amino acid sequence in a 9-mer window (the P5 to P4’ positions in protease nomenclature) determine the cleavage rate at the P1 position, (2) a local response function describing the saturation of the cleavage rate for a single P1 position, (3) a global response

function that determines $K_{50,U}$ based on the sum of the cleavage rates at all possible P1 positions in the full sequence.

To fit the PSSM, we assumed an identical normal prior distribution of scores at all positions, with several exceptions. Due to known critical importance of the P1 position, we used a wider prior distribution of scores for all amino acids in the P1 position for both proteases. We also used wider prior distributions at all positions (P5-P4') for the amino acids Asp, Glu, and Pro, due to the established large effects of these amino acids on cutting rates.

For the local response function to saturation of the cleavage rate at P1 site *k*, we used a logistic function:

$$SS_k = logistic\left(\sum_{site=P5}^{P4'} PSSM(aa_{site}, site)\right) \quad (9)$$

where SS_k (site saturation) is the saturation of the cutting rate at site P1=*k*, aa_{site} is the amino acid identity at *site*, and *logistic* is the logistic function $f(x) = 1 / (1+e^x)$. We fit the 21 (20 amino acids + 'X' representing empty sites) x 9 =189 elements of the PSSM for each protease.

For the global response function (determining $K_{50,U}$ based on the sum of SS_k across the full protein sequence), we use a sum of logistic functions with 10 different activation thresholds.

$$K_{50,U} = maxK_{50,U} - Scale * \sum_{l=1}^{10} logistic\left(\left(\sum_{k=1}^{length\ of\ seq} SS_k\right) - threshold_l\right) \quad (10)$$

where $maxK_{50,U}$ is the highest possible $K_{50,U}$ value ($K_{50,U}$ assuming no cut sites), *Scale* is the range of possible $K_{50,U}$ values, and $threshold_l$ is the value of the lth activation threshold for the global response function. All K_{50} values (including $maxK_{50,U}$) are in $\log_{10}$ molar units.

The key parameters of the unfolded state model (for a single protease) are the 21 x 9 =189 elements of the *PSSM*, the $maxK_{50,U}$, the *scale*, and the 10 *threshold* values. These parameters determine $K_{50,U}$ for each sequence by Eqs. 9 and 10. In addition to these parameters, we also sample the ΔG values for each scrambled sequence during fitting. These sampled parameters (as well as the universal $K_{50,F}$ value for all sequences) are sufficient to determine a theoretical K_{50} value for each scrambled sequence by re-writing Fig. 1b Eq. 6:

$$1/K_{50} = \frac{([U]/K50_u) + ([F]/K50_f)}{([U]+[F])} = f_U/K_{50,U} + (1 - f_U)/K_{50,F} \quad (11)$$

where f_U is the fraction of unfolded molecules:

$$f_U = 1/(1 + exp(\frac{\Delta G}{RT})) \quad (12)$$

The input data for the model are the observed K_{50} values for all scrambled sequences. The parameters of the model are fit by assuming that all observed K_{50} values should agree (with small, normally distributed errors) with the theoretical K_{50} values determined by the model parameters. After fitting the model, we used the median of the posterior distributions of *PSSM*, $maxK_{50,U}$, *scale*, and the 10 *threshold* parameters as the final model parameters. We used these final model parameters to calculate $K_{50,U}$ for all sequences in our experiments without considering any uncertainty from the model posterior distribution.

Procedure for fitting all data

Step 1: Estimation of 'effective' protease concentrations for each library: We employed four DNA oligonucleotide libraries for this study. Although we tried to minimize the difference between assay conditions, we also fit "effective" protease concentrations to our data in order to minimize batch-to-batch differences. We used the K_{50} model to perform this fitting and fit protease concentrations for trypsin and chymotrypsin entirely independently. The main assumption of this fitting is that each sequence should have the same K_{50} when assayed in different libraries. By enforcing that each sequence had a single K_{50} value regardless of what library it appears in, we calibrated the protease concentrations in each library against each other. Although we did not use universal control sequences in all four libraries, each library contained 1000 to 2000 sequences that overlapped at least one other library in a fully connected graph. Specifically, the library pairs 1+4, 2+4, 3+4, 1+2, and 2+3 each included 1,000 to 2,000 overlapping sequences.

The overall model included 96 experimental conditions (12 protease concentrations per replicate x 2 replicates x 4 libraries; one of the 12 protease concentrations was the fixed "no protease" starting condition). However, each sequence was only present in 48 of the 96 conditions because any individual sequence was only present in two out of the four libraries. The inputs to fit the model were the sequencing counts data, the reaction time (*t*), and the k_{max} constant. Additionally, to set the overall scale of the protease concentration series, we fixed the effective protease concentrations for Library 4 at the expected protease concentrations (i.e. three-fold serial dilutions of 25 μM protease (Replicate 1) or 43.3 μM protease (Replicate 2)). We also fixed all of the starting samples at zero protease. Using these model inputs, we sampled the K_{50} values (one per sequence), the remaining 66 protease concentrations, and the initial sequence distributions $cDNA_0$ (a separate $cDNA_0$ was used for each of the 8 replicates). Normal priors (with lower/upper boundaries for some parameters) covering the range of experimentally relevant values were used for the model parameters. Sampling was performed using the No U-Turn Sampler (NUTS) in Numpyro with 50 steps of equilibration and 25 steps of production. We used the medians of the protease concentrations from our 25 posterior samples as our final calibrated protease concentrations for all further analysis (discarding the uncertainties).

Step 2: Estimation of K_{50} values of scramble sequences: To train the unfolded state model, we need to determine K_{50} values for our scramble sequences, which were included in Library 2. We

used the Independent K_{50} model for this step. The input data were the sequencing counts data from two replicates (i.e. 12 protease concentrations x 2 replicates = 24 data points per sequence), the reaction time (t), the k_{max} constant, and the effective protease concentrations obtained in Step 1. We sampled the initial sequence distribution $cDNA_0$ (a separate $cDNA_0$ for each replicate) and K_{50} for all sequences included in Library 2. Normal priors (with lower/upper boundaries for some parameters) covering the range of experimentally relevant values were used for the model parameters. Sampling was performed using the No U-Turn Sampler (NUTS) in Numpyro with 100 steps of equilibration and 50 steps of production.

Step 3: Construction of unfolded state model: We trained the unfolded state model for predicting $K_{50,U}$ using K_{50} values obtained in Step 2. The input sequences were scrambled sequences of wild-type domains selected for deep mutational screening. In addition to our set of exactly scrambled sequences (matching the wild-type amino acid composition 100%), we also included scrambled sequences containing 50%, 60%, 70%, 80%, and 90% of the number of hydrophobic amino acids in the original wild-type sequences. These sequences helped ensure the large majority of our scrambled pool was fully unfolded. Additionally, because all sequences in our experiments are padded with G/S/A linkers up to a constant length, we generated scrambled sequences using two different padding procedures. In the first approach, we designed scrambled sequences that matched the original wild-type length and were padded with G/S/A up to 72 amino acids. In the second approach, we designed 72 amino acid-length scrambles approximately matching the composition of an original wild-type domain, regardless of the length of that wild-type. These scrambled sequences required no additional padding. After measuring K_{50} for all scrambles, we only used sequences with a 95% confidence interval smaller than 0.5 $\log_{10}$ molar units for model training for model fitting (64,238 sequences in total, see Extended Data Fig. 3). In addition to the exact experimental sequences, we also augmented the training dataset with dummy sequences where GS linkers were replaced by the blank 'X' amino acid.

The inputs for the model are amino acid sequences created as described above, and their observed K_{50} for trypsin and chymotrypsin obtained in Step 2. The parameters of the model are fit by assuming that all observed K_{50} values should agree (with small, normally distributed errors) with the theoretical K_{50} values. In this model, we sampled the 21 x 9 =189 elements of the *PSSM*, the *site bias*, the $maxK_{50,U}$, the *scale*, and the 10 *threshold* values. These parameters determine $K_{50,U}$ for each sequence by Eqs. 9 and 10. In addition to these parameters, we also sample the ΔG values for each scrambled sequence during fitting.

Normal priors (with lower/upper boundaries for some parameters) covering the range of experimentally relevant values were used for the model parameters. Using NUTS model, we sampled the parameters described above, then reported the median of the 100 posteriors after removing the initial 400 steps. In Step 4, we used these final model parameters to calculate $K_{50,U}$

for all sequences in our experiments without considering any uncertainty from the model posterior distribution.

Step 4: Prediction of unfolded K_{50} values ($K_{50,U}$) across the full dataset: Using the final model parameters obtained in Step 3, we predicted $K_{50,U}$ values for each amino acid sequence in the libraries without considering any uncertainty. Additionally, since the model was constructed to predict unfolded K_{50} for sequences with 86 amino acids, we added a Gly linker 'GGG' to both ends, followed by padding by 'X' up to 86 amino acids.

Step 5: Estimation of K_{50} values and calculation of ΔG for trypsin and chymotrypsin: We applied the *Coupled* K_{50} model to each of the four libraries separately. The inputs to the model are the sequencing count data from trypsin and chymotrypsin experiments (i.e. 12 protease concentrations x 2 replicates x 2 proteases = 48 data points per sequence), the effective protease concentrations obtained in Step 1, the reaction time, the k_{max} constant ($t*k_{max} = 10^{0.65}$ based on qPCR analysis; see Extended Data Fig. 1), the $K_{50,F}$ constants (3 for trypsin, 2 for chymotrypsin; determined based on the dynamic range of proteolysis experiment; see Extended Data Fig. 5), and the $K_{50,U}$ values predicted by the unfolded model in Step 4. Using the inputs, we sampled ΔG shared between trypsin and chymotrypsin, and initial sequence distribution $cDNA_0$ for each protease for each replicate (although our experiments utilized the same batch of the cDNA-protein complex for two replicates).

Normal priors (with lower/upper boundaries for some parameters) covering the range of experimentally relevant values were used for the model parameters. Using NUTS in Numpyro module, we sampled the posteriors of shared ΔG along with other parameters, then obtained the median of the 50 posterior samples after removing the initial 100 steps. Full results from both the independent and coupled fitting procedure are provided in Tsuboyama2023_Dataset1_20230416.csv and Tsuboyama2023_Dataset2_Dataset3_20230416.csv. For our stability parameters (protease-specific K_{50} in the independent procedure and ΔG in the coupled procedure) we report the median of the posterior distribution as well as the upper and lower limits of the 95% confidence interval (the 2.5%ile and 97.5%ile values of the posterior distribution).

We also applied the *Independent* K_{50} model to each of the four libraries separately. The inputs to the model are the sequencing count data (i.e. 12 protease concentrations x 2 replicates = 24 data points per sequence), the effective protease concentrations obtained in Step 1, the reaction time, the k_{max} constant ($t*k_{max} = 10^{0.65}$ based on qPCR analysis; see Extended Data Fig. 1). Using the inputs, we sampled K_{50} for each protease, and initial sequence distribution $cDNA_0$ for each protease for each replicate (although we utilized the same batch of the cDNA-protein complex for two replicates).

Normal priors (with lower/upper boundaries for some parameters) covering the range of experimentally relevant values were used for the model parameters. Using NUTS in Numpyro module, we sampled the posteriors of K_{50} for trypsin and K_{50} for chymotrypsin along with other parameters, then obtained the median of the 50 posterior samples after removing the initial 100 steps.

Then, we computed protease-specific ΔG values using the protease-specific K_{50} values from the *Independent* model. We do this using the same $K_{50,F}$ and $K_{50,U}$ values used in the coupled procedure according to Fig. 1b Eqs. 5 and 7. These protease-specific ΔG estimate s are also reported in Tsuboyama2023_Dataset2_Dataset3_20230416.csv, and are only used to examine the consistency between different proteases (e.g. Fig. 1f and Fig. 2d). In some cases, the independently fit K_{50} values can lead to impossible values for ΔG. This can occur if K_{50} is higher than $K_{50,F}$ (observed cleavage is slower than our limit for cleavage in the folded state) or if K_{50} is lower than $K_{50,U}$ (observed cleavage is faster than predicted cleavage in the unfolded state). If the median protease-specific K50 or the confidence interval limits for a particular sequence lead to impossible ΔG values for that sequence, we reported dummy values for the corresponding protease-specific ΔG estimates.

The actual number of sequencing counts, as well as the number of counts predicted for all sequences at all concentrations according to the fitted model parameters, are given in Raw_NGS_count_tables.zip and Pipeline_K50_dG.zip.

Thermodynamic coupling analysis (related to Fig. 4)

Thermodynamic coupling refers to the change in folding stability due to the interaction between two amino acids after removing folding stability effects from each amino acid individually. To determine this "nonadditivity", we first modeled our double mutant data using a fully additive model (no thermodynamic coupling). The deviations from this model then reveal the thermodynamic coupling. Our additive model assumes that the absolute stability (ΔG) of each sequence is the sum of an amino acid-dependent term for site one (ΔG_1) and an amino acid-dependent term for site two (ΔG_2)

$$Expected\,\Delta G_{aa1,aa2} = \Delta G_{1,aa1} + \Delta G_{2,aa2} \quad (13)$$

The forty site-specific terms (one ΔG_1 term for each amino acid at site one and one ΔG_2 term for each amino acid at site two) are not experimentally measurable; they are inferred based on minimizing the error of the additive model. We used Bayesian inference to infer the forty ΔG_1 and ΔG_2 terms for each set of mutants. The inputs to fit the model were the observed 400 ΔG values (20 amino acids at site one x 20 amino acids at site two) for a particular site pair. Using NUTS, we sampled ΔG_1 and ΔG_2 by assuming that the 400 observed ΔG values should agree (with small, normally distributed errors) with the expected ΔG values determined by eq. 13. Both

expected and observed ΔG values were clipped to the range of -1 to 5 kcal/mol. We used 100 steps of burn-in and used the median of 50 posterior samples as the final values of the ΔG_1 and ΔG_2 terms. Using these terms, we calculated the expected (additive) ΔG for each sequence, and then the thermodynamic coupling:

$$Thermodynamic\ coupling_{aa1,\,aa2} = Observed\ \Delta G_{aa1,aa2} - Expected\ \Delta G_{aa1,aa2} \quad (14)$$

To calculate the uncertainty in the thermodynamic coupling, we re-fit the additive model 50 times by bootstrap resampling of the 400 observed ΔG values. This ensures the ΔG_1 and ΔG_2 terms are not overly dependent on a single experimental measurement. The model fitting code is provided in Additive_model_Fig4.ipynb.

Wild-type amino acid prediction model (related to Fig. 5)

The classification model in Fig. 5 used a sum of logistic functions with learned amplitudes to define the weighting function. The overall model is defined below:

$$p(aa) = Softmax\left(\left(\sum_{i=1}^{100} amp_i * logistic\left((\Delta G_{aa} - threshold_i) * steepness\right)\right) + offset_{aa}\right) \quad (15)$$

where *p(aa)* is the probability of amino acid *aa*, *softmax* is the softmax function $\frac{e^{x_{aa}}}{\sum_{aa} e^{x_{aa}}}$, *logistic* is the logistic function f(x) = 1 / (1+e^x), *i* indexes the 100 logistic functions defining the weighting function, *amp* is the learned vector describing the amplitudes of the logistic functions, *threshold* is the vector describing the centers of the logistic functions, *steepness* defines the steepness of the logistic functions, and *offset* the learned vector (length 19 for the 19 non-Cys amino acids) describing the absolute probability offset for each amino acid.

We used Bayesian inference to infer the *amp* vector (length 100) and *offset* vector (length 19 for the 19 non-Cys amino acids). The logistic *threshold* vector was fixed at 100 evenly spaced points between -2 and 7 kcal/mol. The *steepness* term was fixed at 5. The inputs to fit the model were the observed ΔG values and the wild-type amino acid identities for each site within the natural protein domains. Using NUTS, we sampled *amp* and *offset* by assuming that the observed wild-type amino acids were randomly chosen at each site according to the predicted probability distribution for that site, calculated according to eq. 15. We then reported the median and the standard deviation of 100 posterior samples after removing the initial 500 steps. The fitting script is included in Classification_model_Fig5.ipynb.

Derivation of eq.3 in Fig. 1b

We modeled the cleavage events, where Protease enzymes (E) and protein substrates (S) form an ES complex to produce cleaved protein products (P). The goal is to get a product formation

equation in terms of the total product, initial enzyme and substrate concentrations and kinetic constants.

$$[E] + [S] \underset{k_{-1}}{\overset{k_1}{\rightleftharpoons}} [ES] \xrightarrow{k_2} [E] + [P] \quad (1)$$

Also, we defined equilibrium constant K_{50}:

$$K_{50} = \frac{[E][S]}{[ES]} \ (1')$$

Based on the model (1), we can obtain the following dynamic formulas:

$$\frac{d[S]}{dt} = -k_1[E][S] + k_{-1}[ES] \ (16)$$

$$\frac{d[ES]}{dt} = k_1[E][S] - k_{-1}[ES] - k_2[ES] \ (17)$$

$$\frac{d[P]}{dt} = k_2[ES] \ (18)$$

The first two of these are assumed to be at quasi-steady state. The following are additional conservation equations for substrate-product and enzyme:

$[S_0] = [S] + [ES] + [P]$ (19) where [S_0] is initial amount of substrates

$[S_{total}] = [S] + [ES]$ (20)

Additionally, the reaction conditions in the study were not substrate-excessive but enzyme-excessive:

$[E_{total}] = [E] + [ES] \approx [E]$ (21) (because [E] >> [ES] or [S])

Using eqs. 1',19, and 20, the following can be derived to find an expression for the enzyme-substrate complex in terms of the initial substrate and enzyme concentration:

$$[ES] = 1/K_{50}[E][S] = 1/K_{50}[E]\,(S_0 - [ES] - [P])$$

$$[ES](1 + 1/K_{50}[E]) = 1/K_{50}[E](S_0 - [P]) = 1/K_{50}[E][S_{total}]$$

$$[ES] = \frac{1/K_{50}[E]}{1+1/K_{50}[E]}[S_{total}] = \frac{[E]}{K_{50}+[E]}[S_{total}] \ (22)$$

Substituting eq. 22 into eq. 18 and using the approximation$[E_{total}] \approx [E]$, the an expression for the dynamics of the product formation in terms of enzyme concentration and substrate can be found:

$$\frac{d[P]}{dt} = \frac{k_2\,[E_{total}]}{K_{50}+[E_{total}]}[S_{total}]$$

Thus, the observed kinetic rate is $k_{obs} = \frac{k_2[E_{total}]}{K_{50} + [E_{total}]}$ (This eq. 3)

Derivation of eq.6 and eq.7 in Fig. 1b

We modeled the cleavage events, where Protease enzymes (E) and folded substrates (F) or unfolded substrates (U) form a FE or UE complex to produce cleaved protein products (P_F or P_U). The goal is to get a product formation equation in terms of the total product, initial enzyme and substrate concentrations and kinetic constants. We follow a similar derivation to that above for a single enzyme/substrate:

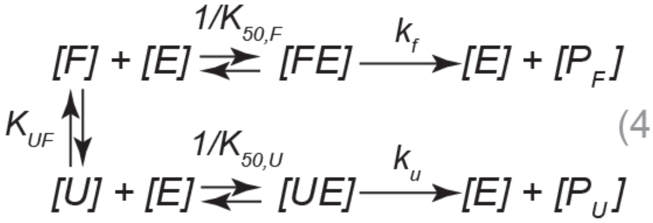

(4)

where $1/K_{50,F} = \frac{[FE]}{[F][E]}$ (23), $1/K_{50,U} = \frac{[UE]}{[U][E]}$ (24), $K_{UF} = \frac{[U]}{[F]}$ (25) , and k_f and k_u are rate constant for cleavage of the bound folded substrates and unfolded substrates. Assuming binding and unbinding equations and the folding and unfolding transition rates are in a quasi-equilibrium then eq 23, 24, and 25 hold throughout the time-course.

We write an equation for the overall product formation:

$\frac{d([P_F]+[P_U])}{dt} = k_f[FE] + k_u[UE]$ (26)

Conservation equations for substrate-product and enzyme in this case are:

$[S_0] = [FE] + [UE] + [F] + [U] + [P_F] + [P_U]$ (27) where $[S_0]$ is initial and total concentration of substrate

$[E_0] = [E] + [FE] + [UE]$ (28) where $[E_0]$ is the initial concentration of enzyme.

Step 1: Write product formation eq. 26 in terms of [FE] and constants only, by substituting for [UE] complex.

$[UE] = 1/K_{50,U}[U][E]$ (use eq. 24)

$= 1/K_{50,U} * K_{UF}[F][E]$ (use eq. 25)

$= \frac{K_{50,F}K_{UF}}{K_{50,U}}[FE]$ (29) (use eq. 23)

$\frac{d([P_F]+[P_U])}{dt} = k_f[FE] + k_u[UE] = (k_f + \frac{K_{50,F}K_{UF}}{K_{50,U}}k_u)[FE]$ (30, use eq. 26)

Step 2: Replace [FE] dependence with ($[S_0]$ - $[P_F]$ - $[P_u]$) dependence using conservation laws

$[S_0] = [FE] + [UE] + [F] + [U] + [P_F] + [P_U]$ (27)

$= [FE] + \frac{K_{50,F}K_{UF}}{K_{50,U}}[FE] + \frac{K_{50,F}}{[E]}[FE] + \frac{K_{UF}K_{50,F}}{[E]}[FE] + [P_F] + [P_U]$

(use eq. 29, 23, and 23+25)

$$= [FE](1 + \frac{K_{50,F}K_{UF}}{K_{50,U}} + \frac{K_{50,F}(1+K_{UF})}{[E]}) + [P_F] + [P_U]$$

Thus, we get an equation which describes the dependence of [FE] on initial substrates and products, with terms in the denominator that capture sequestration in intermediate bound states.

$$[FE] = ([S_0] - [P_F] - [P_U])/(1 + \frac{K_{50,F}K_{UF}}{K_{50,U}} + \frac{K_{50,F}(1+K_{UF})}{[E]})$$

$$= \frac{(S_0 - [P_F] - [P_U])[E]K_{50,U}}{[E]K_{50,U} + K_{50,F}K_{UF}[E] + K_{50,U}K_{50,F}(1+K_{UF})}$$

$$= \frac{(S_0 - [P_F] - [P_U])[E]K_{50,U}}{[E](1/K_{50,F} + K_{UF}/K_{50,U}) + 1 + K_{UF}} \quad (31)$$

Substituting this into the product formation equation:

$$\frac{d([P_F] + [P_U])}{dt} = (k_f + \frac{K_{50,F}K_{UF}}{K_{50,U}}k_u)[FE] \quad (30)$$

$$= (k_f + \frac{K_{50,F}K_{UF}}{K_{50,U}}k_u)\frac{(S_0 - [P_F] - [P_U])[E]K_{50,U}}{[E](1/K_{50,F} + K_{UF}/K_{50,U}) + 1 + K_{UF}} \quad \text{(use eq. 31)}$$

Then, we defined $[P_{total}] = [P_F] + [P_U]$

$$\frac{d([P_{total}])}{dt} = (k_f + \frac{K_{50,F}K_{UF}}{K_{50,U}}k_u)\frac{[E]K_{50,U}}{[E](1/K_{50,F} + K_{UF}/K_{50,U}) + 1 + K_{UF}}([S_0] - [P_{total}])$$

$$= \frac{k_f/K_{50,F} + K_{UF}k_u/K_{50,U}}{1/K_{50,F} + K_{UF}/K_{50,U}} \frac{[E]}{[E] + (1+K_{UF})/(1/K_{50,F} + K_{UF}/K_{50,U})}([S_0] - [P_{total}])$$

Because the reaction conditions in the study were not substrate-excessive but enzyme-excessive (i.e. [E] >> [S] or [ES]), $[E] \approx [E_0]$:

$$\frac{d([P_{total}])}{dt} = \frac{k_f/K_{50,F} + K_{UF}k_u/K_{50,U}}{1/K_{50,F} + K_{UF}/K_{50,U}} \frac{[E_0]}{[E_0] + (1+K_{UF})/(1/K_{50,F} + K_{UF}/K_{50,U})}([S_0] - [P_{total}]) \quad (32)$$

Finally, We can rewrite the product formation eq. 3 in terms of initial substrate concentration, total product, and an observed kinetic rate, which is a function of kinetic rates and initial enzyme concentration,:

$$\frac{d([P_{total}])}{dt} = k_{obs} * ([S_0] - [P_{total}]) = \frac{k_{max}[E_0]}{K_{50} + [E_0]} * ([S_0] - [P_{total}]) \quad (3')$$

Step 3, Derivation eq.6 and eq.7 in Fig. 1b

By comparing eq. 32 with eq. 3', we can derive the following equations (including eq. 6 in Fig. 1B):

$$k_{max} = \frac{k_f/K_{50,F} + K_{UF}k_u/K_{50,U}}{1/K_{50,F} + K_{UF}/K_{50,U}} \quad (33)$$

$$K_{50} = \frac{1+K_{UF}}{1/K_{50,F} + K_{UF}/K_{50,U}} \quad (34)$$

$$= \frac{1 + [U]/[F]}{[FE]/[E][F] + ([U]/[F])*([UE]/[E][U])}$$

$$= \frac{1 + [U]/[F]}{[FE]/[E][F] + [UE]/[E][F]}$$

$$= \frac{([F] + [U])[E]}{[FE] + [UE]} \quad \text{(This is eq. 6)}$$

Using eq. 34 to rewriting a formula for K_{UF} in terms of the half-max reaction rates:

$$K_{50}(1/K_{50,F} + K_{UF}/K_{50,U}) = 1 + K_{UF}$$

$$K_{UF}(K_{50}/K_{50,U} - 1) = 1 - K_{50}/K_{50,F}$$

Thus, eq.7 which gives the ratio of unfolded to folded substrate is derived:

$$\frac{[U]}{[F]} = K_{UF} = \frac{1/K_{50} - 1/K_{50,F}}{1/K_{50,U} - 1/K_{50}} \quad \text{(This is eq. 7)}$$

<u>cDNA display proteolysis protocol</u>

5M NaCl	1.6 µL
1M Tris pH7.4	1.6 µL
cnvK linker	2 µL (20 µM)
RNA	40 pmol
MQ	up to 40 µL

↓

95°C 5 min

Cooled down to 45°C slowly (~0.1 C/s)

Emitted by UV handy lamp 365 nm 6W for ~15 min

↓

In vitro translation by Pure frex 2.0

Sol I	80 µL
Sol II	8 µL
Sol III	16 µL
RNA-cnvK linker duplex	40 µL
RNase inhibitor	4 µL

Add MQ up to 160 µL total

↓ 37°C 2 h

Add 16 µL 0.5M EDTA

Add 160 µL 2x binding Buffer (20 mM Tris pH 7.5, 2 mM EDTA, 2M NaCl, 0.2% Tween)

↓
Add to 200 µL C1 streptavidin beads
↓ 25°C ~20 min
Washed by 1x binding buffer
Washed by Sol B buffer (100 mM NaCl 10 mM Tris 7.5)
Add 400 µL RT solution (PrimeScript RT reagent Kit from TAKARA)

5x RT buffer	80 µL
Enzyme	20 µL
MQ	300 µL

↓ 37°C ~30 min with mix
Washed by His buffer (30 mM Tris pH7.4, 0.5M NaCl, 0.05% Tween)
+ 4 µL RNaseT1 (Thermo) in 400 µL His buffer (without imidazole)
↓ 37°C 15 min with mix
To 800 µL His magnetic beads
↓ 25°C 30 min
Washed by His buffer x3
Add 400 µL 400 mM Imidazole in His buffer
↓ 25°C 15 min
Zeba spin (buffer-exchanged to PBS)
Split into 4 tubes and stocked in -80°C freezer

Add PBS up to 240 µL to thawed protein-cDNA complex
Split into 20 µL x 12 tubes
Add 40 µL protease solution to each tube
↓ RT 5 min
Add 200 µL 2% BSA in PBS
Add 40 µL Dynabeads with 0.66 µg Anti-PA-tag antibody
↓ 4°C 1h
Washed by washing buffer (PBS including 800 mM NaCl and 1% Triton) x3
Washed by PBS x3
↓
Add PA elution buffer 50 µL (250 ug/mL PA peptide, 200 ug/mL BSA in PBS)
↓
Preparing NGS sample
